# Local adaptation in natural European host grass populations with asymmetric symbiosis

**DOI:** 10.1371/journal.pone.0215510

**Published:** 2019-04-17

**Authors:** Päivi H. Leinonen, Marjo Helander, Beatriz R. Vázquez-de-Aldana, Iñigo Zabalgogeazcoa, Kari Saikkonen

**Affiliations:** 1 Natural Resources Institute Finland (Luke), Helsinki, Finland; 2 Department of Biology and Biodiversity Unit, University of Turku, Turku, Finland; 3 Institute of Natural Resources and Agrobiology (IRNASA-CSIC), Salamanca, Spain; 4 Natural Resources Institute Finland (Luke), Turku, Finland; Umeå Plant Science Centre, Umeå University, SWEDEN

## Abstract

Recent work on microbiomes is revealing the wealth and importance of plant-microbe interactions. Microbial symbionts are proposed to have profound effects on fitness of their host plants and vice versa, especially when their fitness is tightly linked. Here we studied local adaptation of host plants and possible fitness contribution of such symbiosis in the context of abiotic environmental factors. We conducted a four-way multi-year reciprocal transplant experiment with natural populations of the perennial grass *Festuca rubra s*.*l*. from northern and southern Finland, Faroe Islands and Spain. We included *F*. *rubra* with and without transmitted symbiotic fungus *Epichloë* that is vertically transmitted via host seed. We found local adaptation across the European range, as evidenced by higher host fitness of the local geographic origin compared with nonlocals at three of the four studied sites, suggesting that selection pressures are driving evolution in different directions. Abiotic factors did not result in strong fitness effects related to *Epichloë* symbiosis, indicating that other factors such as herbivory are more likely to contribute to fitness differences between plants naturally occurring with or without *Epichloë*. Nevertheless, in the case of asymmetric symbiosis that is obligatory for the symbiont, abiotic conditions that affect performance of the host, may also cause selective pressure for the symbiont.

## Introduction

Variability in direction and magnitude of natural selection is a major force shaping biodiversity [1]. As a result, natural populations encountering differing selection pressures become genetically differentiated and locally adapted [2–4]. Local adaptation is traditionally defined as higher fitness of local than nonlocal individuals in a given environment [5]. Selective agents driving local adaptation consist of both abiotic and biotic factors, and the latter become especially apparent when local populations of closely interacting species coevolve [6,7].

Plant-associated symbionts have the potential to be highly beneficial for the fitness of their hosts, as has been shown for nitrogen-fixing rhizobia and mycorrhizal fungi. This makes symbiotic associations between plants and microbes excellent study systems for examining how patterns of local adaptation are shaped by symbiosis, especially because plants as sessile organisms need to adapt to surrounding environmental conditions. Estimating the role of symbiotic associations in local adaptation should involve natural environments, where fitness benefits are determined by resource acquisition and allocation. For example, using local and nonlocal soils and reciprocal inoculation, it has been shown that local soil and local genotypes of arbuscular mycorrhizal fungi promote resource acquisition on *Andropogon gerardii* [8]

At its most extreme, coevolution of hosts and symbionts can result in obligatory associations where survival or reproduction are not possible without the symbiotic partner, and fitness of the symbiont and the host become tightly linked. In these cases, selection against nonlocal host plants results in potential fitness reduction for the symbiont. However, the role of vertically transmitted symbionts in local adaptation of their hosts to abiotic environment is still largely unknown. Systemic fungal symbionts of grasses of the genus *Epichloë* (Ascomycota; Clavicipitaceae) are an example of asymmetric interactions, where the fungus grows asymptomatically between host cells inside aboveground tissues of the plant. *Epichloë* reproduces asexually by growing hyphae in newly produced tillers and seeds of the host grass, resulting in vertical transmission, and making them entirely dependent on their host [9]. *Epichloë* species are specialized symbionts of grasses with a shared coevolutionary history with their hosts and are transmitted in host maternal lines [9–11]. In agricultural grasses, *Epichloë* have been viewed as mutualists mostly due to the herbivore-deterring alkaloids that they produce [12]. Studies on natural populations have shown that asymmetric symbiosis that is facultative to the host plant can range from mutualistic to parasitic [13]. Harmful effects on the host plant are most evident in sexual strains of *Epichloë* species that produce spore-forming structures called stromata–a condition known as choke disease–that prevents or hampers development of seeds on the host plant [14]. However, even asexual vertically transmitted *Epichloë* species (formerly *Neotyphodium*, [15]) can be harmful to the host if costs of harboring the symbiont exceed the benefits [16–18]. This balance could be altered in novel environments, where allocation of host resources can change and potentially result in costs of harboring *Epichloë* or benefits of increased resistance to abiotic stress.

As symbiosis is obligatory for reproduction and persistence of *Epichloë*, adaptive evolution of both parties is potentially heavily affected by host plant performance. Because of the tight and asymmetric fitness linkage, adaptation of the host plant to local conditions (temperature, precipitation and annual variation in day length) can play an important role in evolution of grass-*Epichloë* symbiosis. Local adaptation in plants is often associated with differentiation in flowering responses to temperature and photoperiod, and responses to these factors can influence potential for vertical transmission via successful seed production, making environmental factors influencing host plant performance indirectly governing also fitness of the symbiont. Local adaptation of the host can therefore be beneficial for the symbiont, but unless *Epichloë* provides fitness benefits for the host or especially if it is costly, plants with *Epichloë* could be selected against.

Natural selection can promote occurrence of *Epichloë* even when the fungus is not transmitted to all offspring if patterns of selection vary in heterogeneous environments [13,19]. Natural grass populations have been found to consist of plants with and without *Epichloë* at variable frequencies and they might be completely absent in some areas [20–22]. This is in part due to often incomplete vertical transmission, resulting in tillers and seedlings without *Epichloë* even when associations are mutualistic [23,24]. Loss of the symbiont can be associated with absence of selective advantage and potentially also from genetic mismatches between host and symbiont that can arise from evolutionary conflicts between reproductive modes and genetic variation. These conflicts could be prevalent when cross-pollination of flowers introduces new host genotype combinations in seeds that can prevent growth of the vertically transmitted *Epichloë* species that cannot actively choose their hosts [25].

We used natural populations of an outcrossing perennial grass, *Festuca rubra* L. *sensu lato* (Poaceae, red fescue) and its symbiont *Epichloë festucae* (Leuchtm., Schardl, & Siegel), as a model to study local adaptation in host plants and whether naturally occurring plants with or without symbiont show different fitness responses. Classical reciprocal transplant experiments where individuals from different environments are reciprocally transplanted in native environments of each origin allows to test for local adaptation, evidenced by higher fitness of the local population compared with each of the nonlocal populations [5]. To our knowledge, few reciprocal transplant studies with multiple sites spanning a large geographic area have been conducted–especially in the context of how fitness of the host can be modulated by the symbiont at native sites of natural host populations in the field. Our prediction was that local host populations have become locally adapted and host genotypes naturally harboring *E*. *festucae* (referred to as *Epichloë* from here on) could have reduced fitness due to costs of symbiosis, increased fitness due to resistance to abiotic stress or show no differences related to the tested abiotic environments when compared with naturally *Epichloë*-free genotypes. Although positive and negative effects of fungal symbionts including *Epichloë* on growth and reproduction, photosynthetic rate, abiotic stress tolerance, and competitive ability have been documented [26,27], most of these studies have been conducted with cultivars or in agricultural, nutrient-rich environments or greenhouse conditions [28–30]. Use of natural populations and environments can demonstrate ecologically relevant fitness differences, and whether hosts harboring the symbiont are favored by selection in nature.

We conducted a four-way reciprocal transplant experiment across a broad geographic scale in Europe (northern Finland, Faroe Islands, southern Finland and Spain) and estimated fitness by quantifying several fitness components over three years at each site. We aimed at answering the following questions: first, do we find evidence for local adaptation of the host on abiotic environments on a large geographic scale? Our hypothesis was that in a reciprocal transplant experiment in home environments of each geographic origin in the field, local plants would have higher fitness than nonlocals, and tested this hypothesis both at the level of estimated cumulative fitness and individual fitness components (survival, biomass, flowering propensity and number of flowering culms) in each year. Second, how does *Epichloë* symbiosis contribute to host plant fitness in natural environments? More specifically, we tested whether naturally occurring host genotypes with or without *Epichloë* show different fitness responses in local or novel abiotic environments.

## Materials and methods

### Study system

*F*. *rubra s*.*l*. (referred to as *F*. *rubra* from here on) is an outcrossing, perennial fine-leaved, cool-season tuft grass distributed across the Northern Hemisphere. It grows in oligotrophic, mesotrophic and saline habitats with low or moderate levels of competition such as riverbank meadows, semiarid grasslands, rocky outcrops and sea cliffs and it can be found also in harsh arctic habitats as well as alpine meadows. The species has commercial value, as it is one of the most important turf grasses. Natural populations of *F*. *rubra* include plants with variable ploidy levels from tetraploid to octoploid [22]. The proportion of plants with the *Epichloë* symbiont varies between *F*. *rubra* populations, with no *Epichloë* in some regions [20, 22, 31–33].

### Sampling locations and plant material

**Fig 1:**
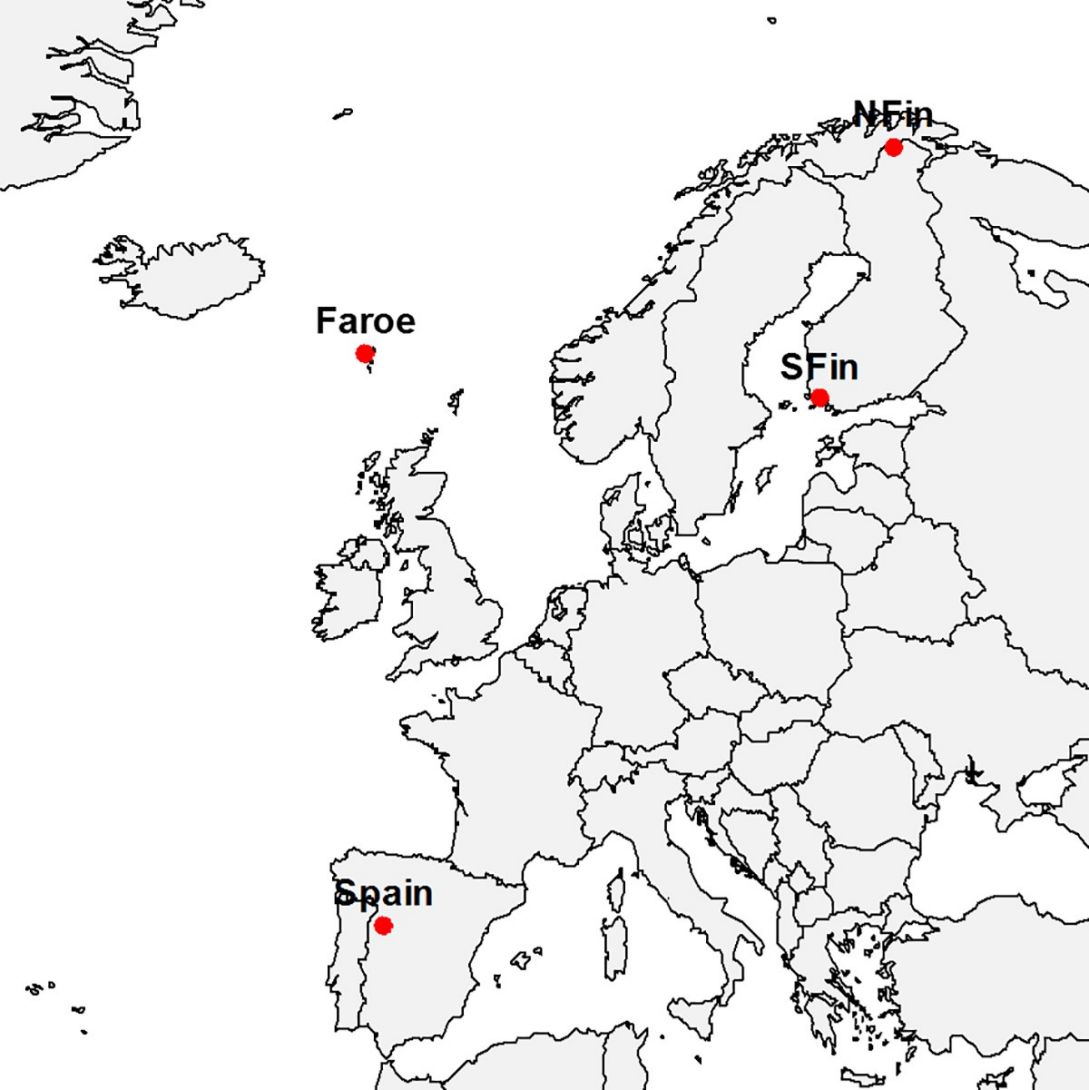
Sampling sites and experimental locations. Locations of the reciprocal transplantation and sampling sites of natural populations of *Festuca rubra* with or without symbiotic fungus *Epichloë festucae* used in this study.

Tillers of *F*. *rubra* were collected from four geographic regions across Europe: northern Finland, Faroe Islands, southern Finland and western Spain (Fig 1, [22]). The plants were not collected from areas requiring permits. In northern Finland, collection sites were two subarctic meadows in Utsjoki, in Finnish Lapland (MS1K: 69° 38' 5.6'' N, 27° 5' 0.9'' E; MS2K: 69° 43' 56.4'' N, 27° 1' 11.6'' E), where the growing season is very short, and winters are long and cold. At the Faroe Islands, where the climate is cool and oceanic with abundant rainfall year around, plants were collected from the islands Mykines (FAS1: 62° 5' 50.7'' N, 7° 40' 55.9'' W) and Viðoy (FAS2: 62° 22' 3.4'' N, W 6° 32' 31.8'' W). In southern Finland, the three sampled populations originated from natural meadows near the shore of the Baltic Sea in Hanko (HA1: 59° 50' 27'' N, 23° 13' 15'' E; HA2: 59° 50' 23'' N, 23° 13' 40'' E; HA3: 59° 53' 0'' N, 23° 5' 52'' E). In Spain, two of the populations were from semiarid oak grasslands (dehesas) near Salamanca (SPLV: 40° 56' 20.16'' N, 6° 7' 6.6'' W; SPPOR: 40° 58' 24.28'' N, 5° 57' 33.69'' W) and one from a Mediterranean oak forest in Garganta de los Infiernos (SPGD: 40° 12' 1.12'' N, 5° 45' 11.03'' W).

To reduce maternal effects prior to the experiment, all field-collected plants were grown in pots filled with a mixture of peat and sand at the Turku University greenhouse in Ruissalo, Finland, where the plants produced new tillers in a common environment. Tillers representing random genotypes from each region (n_N Finland_ = 40, n_Faroe_ = 34, n_S Finland_ = 31, n_Spain_ = 39; S1 Table) were then split to obtain vegetative clones (up to four replicates of each genotype to be planted at each site). The tillers were pre-grown in cell pots (3 cm diameter) for 2–4 weeks prior to planting.

Presence of *Epichloë* in each plant was determined and have been documented earlier based on observations of hyphal growth from surface-sterilized leaf cuttings plated on 5% potato dextrose agar on petri dishes [22]. For estimating performance of natural host genotypes, approximately equal numbers of genotypes with and without *Epichloë* from each region were included, except from southern Finland where none of the plants had *Epichloë* (S1 Table). We did not use plants with manipulated *Epichloë* status, because our aim here was to study effects on natural genotype combinations. *Epichloë* status of plants was verified by spot checks during the experiment.

Examination of ploidy levels in the previous study with flow cytometry showed that most of the Spanish plants included in our study were tetraploid and nearly all genotypes from all other regions were hexaploid [22]. As there were only two octoploid genotypes from northern Finland and three from Spain, and one tetraploid genotype from southern Finland and the Faroe Islands, we were not able to include ploidy level information in our statistical analyses.

### Reciprocal transplant experiment

To estimate fitness in native environments of each region of origin, four reciprocal transplant sites were established in 2012 in home environments of each origin: (i) Kevo Subarctic Research station in Utsjoki in northern Finland (69°45' N, 27°01' E); (ii) The Agricultural Centre (Búnaðarstovan) at the Faroe Islands (62°06' N, 6°57' W); (iii) experimental field in Turku University botanical garden at Ruissalo in southern Finland (60°26' N, 22°10' E) and (iv) at Muñovela Research Farm of IRNASA-CSIC in Salamanca, Spain (40° 54’ N, 5°46’W) (Fig 1). To reduce effects of environmental variation within sites, planting was done in a fully randomized design at each site. Because *F*. *rubra* occurs naturally in relatively competition-free habitats, competing vegetation was removed periodically throughout the course of the experiment. Experimental areas were fenced to exclude large vertebrate herbivores.

Long term climatic observations from weather stations near each transplantation site show differences among sites (Table 1). In general, the growing season is very short at the northernmost site and limited by winter frost and snow. The Faroe Islands are very humid and temperatures are mild year-round. Temperatures in southern Finland are clearly higher than in northern Finland and the growing season is longer, but the climate is more continental than at the Faroe Islands. Mediterranean climate in Spain is characterized by warmer temperatures and the growing season is limited by dry and hot summers. In addition, soil samples were collected at each transplantation site in June 2014 by sampling 3 cm diameter soil cores (depth 0–5cm) which were analyzed for nitrogen, carbon, phosphorus, potassium, calcium and magnesium content as well as pH by Eurofins Viljavuuspalvelu Oy (www.eurofins.fi). Soil from northern Finland and Faroe Islands was found to be more acidic and had higher N and C and lower P, K, Ca and Mg contents than soils from southern Finland and Spain (Table 1).

**Table 1:** Characteristics of climates based on long-term observations at nearby weather stations and soil properties at the sites of transplantation used in this study.

Site	Mean annual temperature°C	Mean temperature in January / July°C	Mean annual precipitation (mm)	Mean precipitation in January / July (mm)	N%	C%	pH	P (mg/liters of soil)	K (mg/liters of soil)	Ca (mg/liters of soil)	Mg (mg/liters of soil)	Ca/Mg
N Finland (Kevo)	-1.7 ^a^	-13.8 / 12.5 ^a^	510 ^a^	34.5 / 75 ^a^	0.17	2.8	5.6	13.4	26.3	1242.2	136.5	9.8
Faroe Islands (Vágar)	6.0 ^b^	2.7 / 10.2 ^b^	1555 ^b^	163 / 115 ^b^	0.23	3.6	5.7	2.7	57.2	937.6	130.6	7.2
S Finland (Turku)	5.7 ^a^	-4.0 / 17.5 ^a^	687 ^a^	54.4 / 77.6 ^a^	0.15	1.5	6.8	10.5	198.4	2696.0	313.8	8.6
Spain (Salamanca)	11.5 ^c^	3.6 / 20.7 ^c^	441 ^c^	39.6 / 10.7 ^c^	0.10	1.1	6.9	39.1	187.4	2231.0	392.4	5.7

^a^Finnish Meteorological Institute (FMI), Utsjoki and Turku 1981–2010

^b^Danish Meteorological Institute (DMI), Vagá Airport, temperature data 1961–1990 precipitation data 1988–1997

^c^ Meteorological station at the planting site in Muñovela experimental farm, Salamanca, Spain 1981–2010.

### Phenotypic measurements

To estimate fitness, data on multiple fitness components was recorded over several years at the four transplantation sites. Survival and flowering status of each plant was determined at each site in three years (2013–2015). Survival in the fourth year at the Spanish site in 2016 was also recorded. To estimate reproductive output at the end of the growing season, the total number of flowering culms was counted for each plant in the three study years (2013–2015) in northern and southern Finland and the Faroe Islands and in 2013–2014 in Spain. To quantify aboveground biomass, the plants were cut at 2–3 cm above the soil surface and weighed at the end of the growing season in 2013 and 2014.

### Statistical analysis

To estimate total fitness, cumulative survival at the end of the experiment was calculated to be able to test for differences in long-term survival. For comparing reproductive fitness over several years, cumulative reproductive success (cumulative number of flowering culms) over three years was used for the sites in northern Finland, Faroe Islands and southern Finland and over two years in Spain, by also including value zero for plants that were not alive or did not flower in each year. Pairwise tests for local adaptation and fitness effects of the presence of *Epichloë* at each site were conducted for both estimates of total fitness. Counts of live/dead or flowering/vegetative for each genotype (1–4 per site) at each site were used as response variables for survival and flowering propensity and for all other traits genotypic means at each site were used.

Likelihood ratio tests (two-tailed) between generalized linear models in R 3.4.1. [34] were used for all statistical comparisons. For modeling survival and flowering propensity, binomial distribution with a logit link function was used, and Gamma distribution with log link for number of flowering culms and biomass. Gaussian distribution was used for cumulative reproductive success with log10+1 transformation. Model fit was visually inspected using diagnostic plots of residuals.

We identified which fitness components and years show different responses depending on region of origin or presence of *Epichloë* (present or absent) across sites (three-way interactions between site, region of origin and presence of *Epichloë* and their two-way interactions) and included population nested within region as fixed covariate to control for between-population variation within regions. Year was not included as a variable in the models due to lack of degrees of freedom, but data was instead analyzed separately for each year, as fitness effects of *Epichloë* symbiosis have been found to vary between years in earlier studies [25]. These tests were not performed on the number of flowering culms in the 2^nd^ and 3^rd^ year due to low sample size (≤ 5) at some sites when only few individuals flowered. Based on these global test results, we proceeded to test for local adaptation and effect of *Epichloë* symbiosis on fitness.

Cases with significant interaction between region of origin and site were selected for specific pairwise testing for local adaptation. Tests for local adaptation were done according to the ‘local vs foreign’ criterion [5] by performing pairwise comparisons between the local and each of the nonlocal geographic origins at the region level (populations from northern Finland, Faroe Islands, southern Finland and Spain each combined as one region) at each site. For these tests, the models we compared differed only in that they had the regions of origin for the pair to be tested merged in one model and all regions defined separately in the full model and included *Epichloë* status as a covariate in all models. In this way, the likelihood ratio models tested whether categorizing the two regions of origin separately has a significant effect.

Significance of fitness differences between genotypes with or without *Epichloë* at each site were tested in cases when any of the factors involving the presence of *Epichloë* were significant. In these analyses, plants from southern Finland were excluded, as none of them had *Epichloë*. These tests were done by comparing a model with *Epichloë* presence as a fixed effect with a null model separately for each region of origin at each site.

## Results

### Local adaptation

Analysis of fitness data collected at the four transplantation sites showed putative cases for local adaptation indicated by significant region of origin x site interactions in all fitness components in all years (Table 2). More specifically, we found that host plants from the sampled geographic regions showed different fitness responses depending on planting site (significant site x region of origin interactions) in survival, aboveground biomass and flowering propensity in each year, and number of flowering culms in the first year (Table 2). Descriptive statistics and sample sizes for plants from each geographic region planted at each site can be found in supplementary tables for tests of local adaptation (S2 Table) and presence of *Epichloë* (S3 Table).

**Table 2:** Results of statistical comparisons of fitness of *Festuca rubra* with and without symbiont *Epichloë festucae* from northern Finland, Faroe Islands, southern Finland and Spain using likelihood ratio tests between generalized linear models in R testing for interactions between planting site, region of origin and presence of *Epichloë* (symbiont status) and main effect of *Epichloë* on fitness components in reciprocal transplant experiment at the native sites of each geographic region. Main effects or lower order interactions were not tested in cases where a higher order interaction was significant.

	Year 1			Year 2			Year 3		
Fitness component	df	Deviance	P	df	Deviance	P	df	Deviance	P
Survival									
Site x region x *Epichloë*	6	5.19	0.5202	6	5.14	0.5259	6	7.38	0.2875
Site x region	**9**	**47.73**	**< 0.0001**	**9**	**33.26**	**0.0001**	**9**	**21.05**	**0.0124**
Site x *Epichloë*	3	2.55	0.4657	3	1.43	0.6996	3	1.94	0.5856
Region x *Epichloë*	2	0.64	0.7277	2	3.06	0.2161	2	3.05	0.2180
*Epichloë*	1	2.42	0.1200	1	3.83	0.0503	1	3.02	0.0825
Biomass									
Site x region x *Epichloë*	6	7.47	0.1059	6	8.50	0.2460	.	.	.
Site x region	**9**	**54.79**	**< 0.0001**	**9**	**102.48**	**< 0.0001**	.	.	.
Site x *Epichloë*	3	1.75	0.4966	3	3.03	0.4322	.	.	.
Region x *Epichloë*	2	0.93	0.5301	**2**	**7.01**	**0.0421**	.	.	.
*Epichloë*	1	0.90	0.2688	.	.	.	.	.	.
Flowering propensity^a^									
Site x region x *Epichloë*	6	2.94	0.8169	6	9.65	0.1401	6	12.12	0.0593
Site x region	**9**	**93.73**	**< 0.0001**	**9**	**100.58**	**< 0.0001**	**9**	**116.29**	**< 0.0001**
Site x *Epichloë*	3	0.13	0.9883	3	**12.64**	**0.0055**	3	1.17	0.7603
Region x *Epichloë*	2	2.05	0.3596	2	4.22	0.1211	2	4.53	0.1037
*Epichloë*	1	0.98	0.3230	.	.	.	1	2.51	0.1130
N flowering culms									
Site x region x *Epichloë*	6	3.77	0.2939	.	.	.	.	.	.
Site x region	9	**35.91**	**< 0.0001**	.	.	.	.	.	.
Site x *Epichloë*	3	1.02	0.5797	.	.	.	.	.	.
Region x *Epichloë*	2	0.74	0.4924	.	.	.	.	.	.
*Epichloë*	1	0.62	0.2760	.	.	.	.	.	.

**Fig 2:**
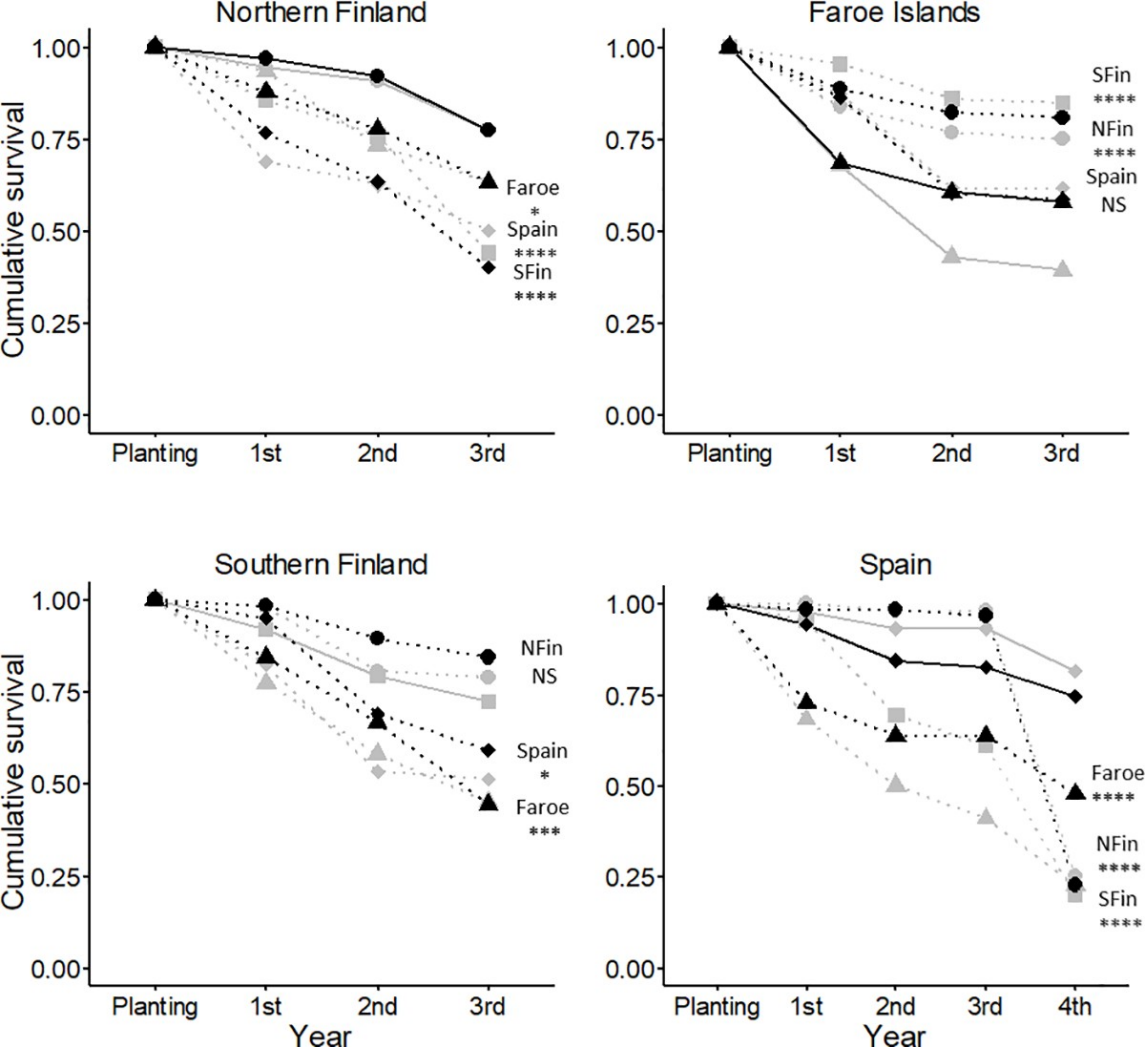
Survival in the field. Cumulative survival of the host plant *Festuca rubra* originating from four geographic regions (circles: northern Finland, triangles: Faroe Islands, squares: southern Finland, diamonds: Spain) for plants with (black) and without (grey) fungal symbiont *Epichloë festucae* in a four-way reciprocal transplant experiment in their local environments in northern Finland, Faroe Islands, southern Finland and Spain. Local geographic origin at each site is indicated with a solid line. Asterisks indicate significance of likelihood ratio tests for survival at the end of the experiment between local and nonlocal genotypes (**** P < 0.0001; *** P < 0.001; ** P < 0.01; * P < 0.05; NS P > 0.05). See Table 3 for full details of results of the statistical tests.

Cumulative survival at the end of the experiment was higher for local plants compared with nearly all the nonlocal origins at three of the studied sites, supporting the local adaptation hypothesis (Fig 2, Table 3). In northern Finland, where 77% of the local plants had survived by the end of the experiment, only 63% of the plants from the Faroe Islands and 44% of plants from both southern Finland and Spain were still alive. A contrasting pattern was seen at the Faroe Islands, where the plants from northern and southern Finland had significantly higher survival by the end of the experiment (78% and 85%, respectively) when compared with survival of local plants (50%). In southern Finland, cumulative survival of plants from the Faroe Islands (45%) and Spain (56%) was significantly lower than survival of the local plants at the same site (72%). In Spain, cumulative survival of the local Spanish plants (78%) was higher than that of the nonlocals from northern Finland (24%), southern Finland (20%) and Faroe Islands (39%). Comparing separate years showed that survival differences accumulated over all study years in northern Finland and in Spain (Fig 2, Table 3). At the Faroe Islands, higher survival of nonlocal plants compared with the locals was due to differences in the first two study years (Fig 2, Table 3). In southern Finland, low survival of the nonlocal Faroese plants was only seen in the first year, while high mortality among the Spanish plants at that site occurred only in the second year (Fig 2, Table 3).

**Table 3:** Tests for local adaptation in *Festuca rubra* based on results of likelihood ratio tests between generalized linear models in R for fitness estimated in reciprocal transplant experiment at native sites of regional origins in northern Finland, Faroe Islands, southern Finland and Spain for estimates of cumulative fitness and fitness components. Significant differences supporting local adaptation (local has higher fitness than nonlocal) are marked with plus signs (+) and cases for local maladaptation (local has lower fitness than nonlocal) with minus signs (-).

	Transplantation site											
	N Finland			Faroe Islands			S Finland			Spain		
Fitness estimate and region of origin	Deviance	P		Deviance	P		Deviance	P		Deviance	P	
Cumulative survival												
N Finland				15.24	< 0.0001	-	1.33	0.2492		61.59	< 0.0001	+
Faroe Islands	4.35	0.037	+				13.28	0.0003	+	24.49	< 0.0001	+
S Finland	22.98	< 0.0001	+	22.04	< 0.0001	-				49.55	< 0.0001	+
Spain	26.00	< 0.0001	+	1.70	0.1927		6.57	0.0104	+			
Cumulative reproductive output												
N Finland				13.73	0.0002	-	9.01	0.0146	+	0.19	0.7506	
Faroe Islands	34.63	< 0.0001	+				46.20	< 0.0001	+	106.10	< 0.0001	+
S Finland	9.43	< 0.0001	+	20.83	< 0.0001	-				18.29	0.0023	+
Spain	44.60	< 0.0001	+	15.32	< 0.0001	-	18.87	0.0005	+			
Survival (1^st^ year)												
N Finland				8.56	0.0034	-	2.47	0.1161		2.48	0.1153	
Faroe Islands	2.54	0.1109					7.00	0.0082	+	18.86	< 0.0001	+
S Finland	4.77	0.0289	+	19.09	< 0.0001	-				0.06	0.8059	
Spain	23.74	< 0.0001	+	8.35	0.0039	-	1.65	0.1989				
Survival (2^nd^ year)												
N Finland				5.68	0.0172	-	0.11	0.7437		6.54	0.0105	-
Faroe Islands	6.23	0.0125	+				3.29	0.0697		2.70	0.1005	
S Finland	2.87	0.0901		4.49	0.0341	-				7.67	0.0056	+
Spain	5.16	0.0232	+	0.82	0.3641		9.26	0.0023	+			
Survival (3^rd^ year)												
N Finland				0.95	0.3287		3.04	0.0813		0.03	0.8545	
Faroe Islands	0.02	0.8777					1.94	0.1632		1.74	0.1877	
S Finland	16.67	< 0.0001	+	1.11	0.291					5.18	0.0229	+
Spain	4.89	0.027	+	1.23	0.2665		0.27	0.6023				
Survival (4^th^ year)												
N Finland										85.38	< 0.0001	+
Faroe Islands										6.23	0.0126	+
S Finland										38.28	< 0.0001	+
Biomass (1^st^ year)												
N Finland				9.34	0.0008	+	1.50	0.1661		0.74	0.2736	
Faroe Islands	0.00	0.9604					0.08	0.7456		12.21	< 0.0001	+
S Finland	5.19	0.0705		8.48	0.0014	+				6.24	0.0018	+
Spain	12.36	0.0056	+	1.00	0.2647		4.76	0.0145	+			
Biomass (2^nd^ year)												
N Finland				11.83	0.0023	+	0.91	0.4498		1.76	0.0366	+
Faroe Islands	7.09	0.0699					7.11	0.0361	-	2.56	0.0121	+
S Finland	8.79	0.0439	+	15.48	0.0005	+				4.68	0.0008	+
Spain	45.60	< 0.0001	+	33.35	< 0.0001	+	59.35	< 0.0001	+			
Flowering propensity (1^st^ year)												
N Finland				3.14	0.0765		0.22	0.637		1.93	0.1646	
Faroe Islands	45.02	< 0.0001	+				21.66	< 0.0001	+	62.71	< 0.0001	+
S Finland	9.97	0.0016	+	1.02	0.3132					5.78	0.0162	+
Spain	59.78	< 0.0001	+	14.09	0.0002	-	0.05	0.8147				
Flowering propensity (2^nd^ year)												
N Finland				0.95	0.3307		40.49	< 0.0001	+	7.03	0.008	+
Faroe Islands	48.73	< 0.0001	+				60.13	< 0.0001	+	22.98	< 0.0001	+
S Finland	7.95	0.0048	+	1.83	0.1766					1.47	0.2249	
Spain	52.39	< 0.0001	+	2.43	0.1193		16.73	< 0.0001	+			
Flowering propensity (3^rd^ year)												
N Finland				7.16	0.0075	-	12.41	0.0004	+	27.32	< 0.0001	+
Faroe Islands	26.29	< 0.0001	+				9.39	0.0022	+	56.81	< 0.0001	+
S Finland	6.79	0.0092	+	10.81	0.001	-				5.30	0.0214	+
Spain	25.26	< 0.0001	+	8.33	0.0039	+	6.85	0.0089	+			
Number of flowering culms (1^st^ year)												
N Finland				0.17	0.5422		2.92	0.0261	+	5.60	0.0036	+
Faroe Islands	2.89	0.0208	+				12.11	< 0.0001	+	12.16	< 0.0001	+
S Finland	1.23	0.1291		0.65	0.2279					22.51	< 0.0001	+
Spain	2.08	0.0488	+	5.86	0.0004	-	0.88	0.2201				

**Fig 3:**
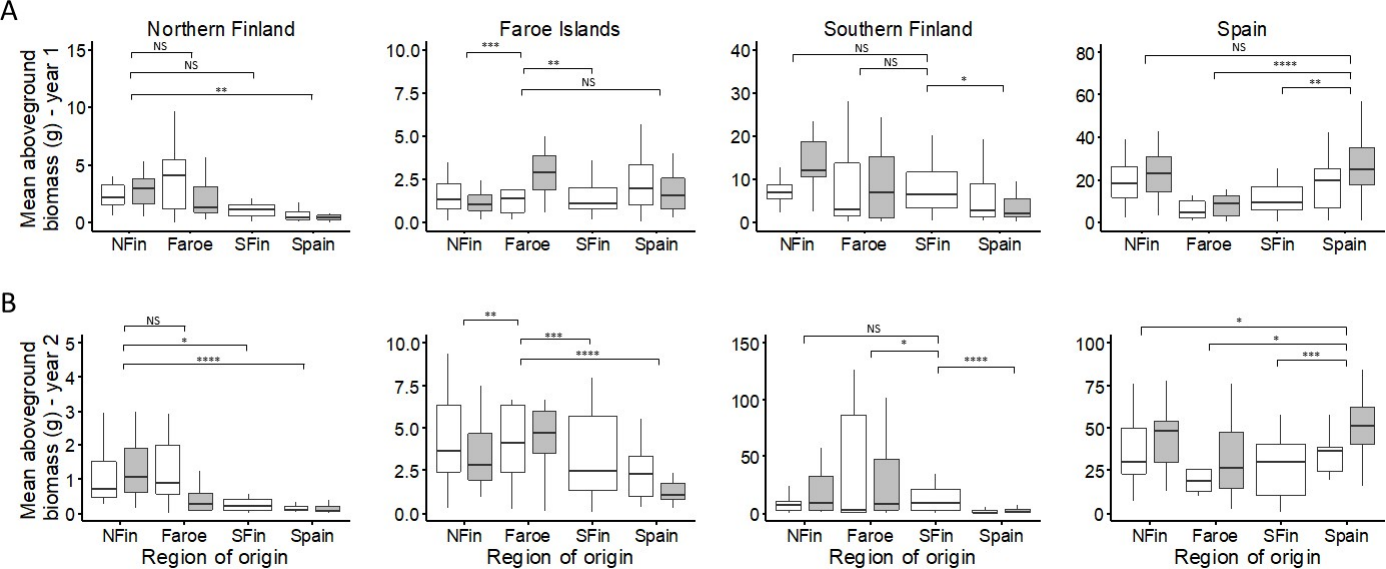
Biomass in the field. Distribution of genotypic means for production of aboveground biomass (g) for the host plant *Festuca rubra* from northern Finland, Faroe Islands, southern Finland and Spain in the first (A) and second (B) year in northern Finland, Faroe Islands and southern Finland and Spain in a four-way reciprocal transplant experiment in local environments of geographic origins (white = plants without *Epichloë*, grey = plants with *Epichloë*). Note the different scale on the y axis. Horizontal line: median, box: first and third quartiles, whiskers: 1.5 * inter-quartile range, outliers not shown. Asterisks indicate significance of likelihood ratio tests between local and nonlocal genotypes (**** P < 0.0001; *** P < 0.001; ** P < 0.01; * P < 0.05; NS P > 0.05). See Table 3 for full details of results of the statistical tests.

Analysis of aboveground biomass production showed evidence for local adaptation at all four sites (Fig 3, Table 3). In general, all the plants were substantially larger at the site in Spain already in the first year than at any other site (Fig 3, S1 Fig). At the site in northern Finland, nonlocal plants from Spain produced less than half the amount of aboveground biomass of the local population in each year (mean ± SD) (1^st^ year: local 2.99 ± 3.05 g vs Spanish 1.15 ± 2.91 g; 2^nd^ year: local 1.50 ± 2.32 g vs Spanish 0.26 ± 0.60 g). Also in the second year, plants from southern Finland produced about a half of the biomass when compared with the locals (0.61 ± 1.65 g). Evidence for local adaptation was also found at the Faroe Islands, where the local plants produced twice as much biomass in the first year (3.24 ± 4.95 g) as plants from northern (1.53 ± 1.50 g) and southern Finland (1.38 ± 1.68 g). This difference was even greater in the second year, when the locals produced up to four times as much biomass (12.47 ± 22.22 g) as the plants from northern (5.66 ± 8.40 g) and southern Finland (3.91 ± 5.06 g) and Spain (2.59 ± 4.03 g), and the differences were significant. In southern Finland, the Spanish plants had significantly lower biomass than the local plants both in the first (local 7.73 ± 7.65 g vs Spanish 4.61 ± 6.58 g) and in the second year (local 14.36 ± 20.92 g vs Spanish 2.25 ± 3.47 g). Relatively high biomass production of Faroese plants was also found in the second year in southern Finland, where the nonlocal Faroese plants outperformed the locals by producing three times the amount of biomass (43.96 ± 73.18 g). In both years in Spain, biomass production of the local plants (1^st^ year 27.30 ± 26.87 g; 2^nd^ year 55.41 ± 41.21 g) was up to two times the amount produced by the plants from the Faroe Islands (1^st^ year 11.43 ± 16.06 g; 2^nd^ year 36.06 ± 32.97 g) and southern Finland (1^st^ year 11.12 ± 9.59 g; 2^nd^ year 29.68 ± 22.58 g) and plants from northern Finland in the second year (39.42 ± 23.31 g).

**Fig 4:**
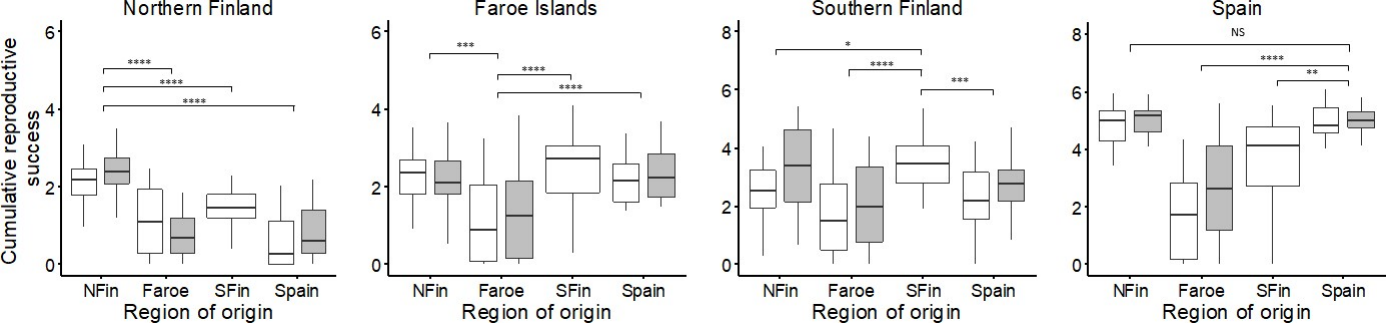
Reproductive fitness. Distribution of genotypic means for cumulative reproductive success of the host plant *Festuca rubra* from northern Finland, Faroe Islands, southern Finland and Spain over three years in northern Finland, Faroe Islands and southern Finland and over two years in Spain in a four-way reciprocal transplant experiment in local environments of geographic origins (white = plants without *Epichloë*, grey = plants with *Epichloë*). Note the different scale on the y axis. Horizontal line: median, box: first and third quartiles, whiskers: 1.5 * inter-quartile range, outliers not shown. Asterisks indicate significance of likelihood ratio tests between local and nonlocal genotypes (**** P < 0.0001; *** P < 0.001; ** P < 0.01; * P < 0.05; NS P > 0.05). See Table 3 for full details of results of the statistical tests.

Cumulative reproductive output combining survival to flowering and number of flowering culms produced in each year showed evidence for local adaptation in northern and southern Finland and Spain, but not at the Faroe Islands (Fig 4, Table 3). At the site in northern Finland, local plants had up to six-fold the cumulative reproductive output (11.06 ± 11.99) compared to the plants from Faroe Islands (2.66 ± 4.78), southern Finland (4.11 ± 7.03) and Spain (1.77 ± 4.02). In contrast to the local adaptation hypothesis, cumulative reproductive output of the local plants at Faroe Islands (6.35 ± 10.88) was only about a half of that of the nonlocal plants (northern Finnish 10.64 ± 11.87; southern Finnish 16.95 ± 22.01and Spanish 11.65 ± 15.04) and was relatively low also at other sites. Local advantage was found in southern Finland, where locals had up to three times as much cumulative reproductive output (47.37 ± 64.36) as plants from all other regions (northern Finnish 38.31 ± 67.80; Faroese 15.97 ± 42.39 and Spanish 20.30 ± 34.62). Also in Spain, the local plants had four times as high cumulative reproductive output (163.10 ± 122.70) as plants from the Faroe Islands (35.50 ± 85.02) and twice as high as plants from southern Finland (79.76 ± 99.27).

In support of local adaptation, flowering propensity of plants that had survived each year was significantly higher for the local plants when compared with the nonlocals, indicating differences in flowering induction or slower development rate of flowering culms (S2 Fig, Table 3). This was especially evident in northern Finland, where all three nonlocal geographic origins had significantly lower flowering propensity than the locals in all studied years. For example, in the first year, nearly all of the local plants flowered when flowering propensity of plants from other regions was significantly lower (79%, 55% and 48% for the plants from southern Finland, the Faroe Islands and Spain, respectively). On the contrary, at the Faroe Islands site in the first year nearly all (91%) Spanish plants flowered while only 64% of the local plants flowered. In later years, flowering propensity of the Spanish plants decreased drastically at the Faroe Islands and in the third study year only 22% of the Spanish plants flowered, while 52% of the local plants flowered that year. Evidence for local adaptation in flowering propensity was clearer in southern Finland, where significantly more local plants were flowering (89%) than the Faroese plants (50%) in the first year. In the next two years, flowering propensity of the locals significantly exceeded that of the nonlocals in all comparisons. Similarly in Spain, flowering propensity of the locals was significantly higher compared with all or some of the nonlocals in all years.

Production of flowering culms among the plants that flowered showed evidence for local adaptation in the first year at three of the four studied sites (S3 Fig, Table 3). In northern Finland, local plants produced 40–50% more flowering culms (6.72 ± 5.81) than plants from the Faroe Islands (4.49 ± 4.47) and Spain (4.68 ± 5.14). There were no differences in flowering culm production at the Faroe Islands. In southern Finland the local plants had 40% more flowering culms (11.83 ± 7.60) than plants from northern Finland (8.62 ± 7.77) and more than two times as many as the plants from the Faroe Islands (4.52 ± 5.69). Local adaptation was supported also in Spain, as the local plants produced two to four times as many flowering culms (27.09 ± 23.19) as the nonlocal plants (N Finnish14.48 ± 12.01; Faroese 8.73 ± 8.54; S Finnish 7.06 ± 6.54).

### Fitness of host plant genotypes with and without *Epichloë* symbiosis

Performance of host plants with or without *Epichloë* did not show strong differences in fitness comparisons (Table 2). We did find a significant interaction between region of origin and presence of *Epichloë* for biomass in the second year and between planting site and presence of *Epichloë* for flowering propensity in the second year (Table 2).

Pairwise tests showed some significant fitness differences between genotypes with and without *Epichloë* among plants from northern Finland and Faroe Islands, but no such differences were found for the Spanish plants (S4 Table). In northern Finland in the second year, plants without *Epichloë* from the Faroe Islands were twice as large in terms of biomass (without *Epichloë* 1.31 ± 1.64; with *Epichloë* 0.51 ± 0.75; Deviance = 8.05, P < 0.01) (Fig 3) and had more flowering individuals (18%) than plants with *Epichloë* (3%) (Deviance = 4.31, P < 0.05). In southern Finland, there was a three-fold difference in cumulative reproductive output favoring host plant genotypes with *Epichloë* among the plants from the northern Finland region (without *Epichloë* 17.04 ± 28.17; with *Epichloë* 57.38 ± 85.43; Deviance = 6.32, P < 0.05) (Fig 4). In the second year in southern Finland, there was a 2.5-fold difference in biomass production favoring plants from northern Finland with *Epichloë* (without *Epichloë* 8.75 ± 12.44; with *Epichloë* 21.82 ± 32.04; Deviance = 5.62, P < 0.05) (Fig 3). Also, 40% of the *Epichloë*-harboring plants from northern Finland flowered, while the percentage of flowering plants without the symbiont was only 4% (Deviance = 18.37, P < 0.0001). In Spain, cumulative survival at the end of the experiment was significantly higher for Faroese plants with (48%) than without *Epichloë* (23%) (Deviance = 4.01, P < 0.05) (Fig 2).

## Discussion

### Local adaptation across Europe

Our large-scale, multi-year reciprocal transplant experiment revealed local adaptation in *F*. *rubra* across Europe, as evidenced by higher fitness in local plants compared with nonlocals in northern Finland, southern Finland and Spain. These findings demonstrate the role of natural selection in shaping genetic and phenotypic differentiation in this widespread host grass species. Evidence for local adaptation was supported by multiple fitness components and cumulative fitness estimates. Other studies on grassland plants have documented local adaptation across Europe in some but not all studied species [35,36]. In our study case local adaptation of the host can have consequences for evolution of both partners as fungal symbiont *Epichloë* is entirely dependent on the grass. Therefore, local adaptation of the host grass will benefit symbiotic partners, and causes natural selection acting against nonlocal host plant genotypes to also decrease performance of nonlocal *Epichloë* strains.

Comparisons across large geographic distances often show local adaptation and differences in selection pressures (e.g. [37–40]). At higher latitudes, plants need to be adapted to strong seasonal changes in temperature, including long winters with temperatures below freezing and variation in day length and light quality. We found that in northern Finland, fitness advantage of the local origin was due to higher survival and flowering propensity than the nonlocal origins. This could be due to differences in photoperiod responses that are required for flowering induction and preparation for overwintering. In *F*. *rubra* as well as in other perennial grasses, flowering induction occurs in two steps where consecutive periods of short days, cold temperatures (vernalization) and long days are required [41]. It is also possible that floral development is in general slower in plants adapted to a longer growing season, and flowering culms in plants from other geographic origins did not have enough time to develop. Plants originating from northern Finland had surprisingly high performance at all sites, indicating that for example responses to photoperiod did not lower their fitness in nonlocal environments. Long-term survival of these northern genotypes was low for example in Spain, possibly due to drought stress during hot and dry summers. Plants from Spanish semiarid grasslands cope with this situation by means of summer dormancy, but seashore populations of *F*. *rubra* have been found to remain green throughout the growing season [42]. Common garden experiments with pasture grasses have shown that plants from different geographic origins differ in their responses to climatic extremes, such as drought, that are associated with climate change [43, 44].

At the Faroe Islands, local plants were outperformed by nonlocal plants in all fitness components except biomass, but the Faroese plants had relatively low survival and reproductive success also at nonlocal sites. At the Faroe Islands, the surviving local individuals seemed to be able to utilize the long growing season in the cool and humid oceanic climate, resulting in larger biomass compared to nonlocals. Larger vegetative size and low flowering propensity and number of flowering culms could indicate that the Faroese plants differ in their allocation to sexual vs vegetative reproduction, as has been found in sea shore populations of *F*. *rubra* in Spain [42]. It is also possible that strong selective pressures related to for example temperature extremes such as cold winters might not have a large role in shaping Faroese populations. This can also have resulted in presence of maladaptive alleles via gene flow either from other regions or cultivars of the same species. There might also be more fine-scale environmental differences across the Faroe Islands that would be revealed by reciprocal transplantations between the specific islands. Furthermore, as our study focused on large-scale climatic differences in abiotic factors between the regions, inclusion of effect of competition with surrounding vegetation could reveal local adaptation also in Faroese plants, if their higher biomass production would be correlated with better competitive ability. However, as *F*. *rubra* occurs in habitats with relatively low competition, inclusion of a competition treatment would have significantly changed our results.

### Role of *Epichloë* symbiosis in fitness variation of the host

Vertical transmission mode of the symbiont is predicted to be associated with mutualistic interactions [45], predicting that *Epichloë* should be generally promoting fitness of their hosts. This is supported by data from agronomical systems where abundance of nutrients can contribute to beneficial effects of symbiosis to the host, as has been documented for example in perennial ryegrass (*Lolium perenne*) and tall fescue (*Festuca arundinacea*) [46]. In a study with both wild grasses and cultivars of tall fescue, an overall beneficial effect of *Epichloë* was reported in a transplantation experiment, but similarly as indicated in our present study on *F*. *rubra*, fitness effects depended on the environment and host plant genotype and varied between years and fitness components [47]. Herbivory is the most studied factor contributing to evolution of the mutualistic association due to alkaloid compounds produced by *Epichloë* [9]. Fitness benefits for the host grass are determined by resource acquisition and allocation, especially when the symbiont is using resources for production of protective alkaloids requiring nitrogen [48]. In natural populations and environments the defensive role may be more variable and context dependent, as levels of alkaloid production profiles and their success for preventing herbivory can vary [49]. In addition, presence of *Epichloë* can also result in reduced vegetative biomass, as was found for example in a natural population of *Festuca arizonica* in a field experiment [48]. We focused here on the role of large scale abiotic factors driving evolution of local adaptation and found no strong fitness differences between plants with or without *Epichloë*, indicating that herbivory rather than abiotic factors is driving local evolution involved in *Epichloë* symbiosis. However, in some cases novel environmental conditions can induce gain or loss of fitness in plants with *Epichloë* in the studied environments. Loss of fitness in Faroese plants harboring *Epichloë* in our study at the site in northern Finland could be due to breakdown of mutualism in nonnative grass-*Epichloë* genotype combinations in stressful conditions, associated with expression changes in a set of fungal genes involved in enhanced nutrient uptake and degradation [50]. On the contrary, in Spain survival of the same Faroese genotypes with *Epichloë* was improved to some degree. This could potentially result from improved resistance to drought in Faroese plants with *Epichloë* as in Spain plants have to cope with seasonal droughts and with very intensive sunlight year-round. Further studies on drought and salt stress resistance could reveal whether thick and waxy leaves of the Faroese plants with *Epichloë* would confer drought tolerance and enable persistence of green leaves throughout the growing season also in dry habitats such as Spanish grasslands. Plants with *Epichloë* from northern Finland showed a clear increase in reproductive fitness and biomass production compared with plants without *Epichloë* when transplanted in southern Finland where the growing season is longer than in their native environment, although no individuals with *Epichloë* have been found in natural *F*. *rubra* populations in this region.

Fitness comparisons of local host plants genotypes with or without *Epichloë* indicated that abiotic factors did not seem to impose selective pressure on *Epichloë* symbiosis, especially at native sites. In another study with Spanish *F*. *rubra* at the same experimental site in Spain, plants with *Epichloë* had greater phosphorus content than plants without *Epichloë* [51], potentially yielding fitness differences in a longer term. Local grazing pressures by large vertebrate grazers not tested here are likely to contribute more to selective advantage of symbiosis, as *Epichloë* occur at high frequencies at collection sites of the studied geographic origins with heavy grazing in northern Finland (reindeer), Faroe Islands (sheep) and Spain (cattle). Even in the absence of fitness benefits, mathematical models based on metapopulation theory have predicted that vertically transmitted *Epichloë* species can be maintained in populations even in the absence of fitness benefits to the host and when *Epichloë* is not transmitted to all developing seeds [19]. However, as fitness effects of *Epichloë* on *F*. *rubra* have been found to change depending on plant age and from year to year [16, 18, 49, 51], studies examining survival and germination success of seeds with or without *Epichloë* could provide more evidence for selective advantage depending on the environment. Differences in germination success could also contribute to resulting frequencies of *Epichloë* occurrence in grass populations, if seedlings with or without *Epichloë* are more successfully recruited [24].

### Possible fitness consequences for the fungal symbiont

Selective forces driving evolution of the fungal partner *Epichloë* are tightly correlated with host fitness, and persistence and vegetative reproduction of the host enables survival and growth of *Epichloë*. Local adaptation of the host plant in our study has strong implications for fitness of the fungal symbiont *Epichloë*, as nonlocal fungal genotypes are selected against when survival of the nonlocal hosts is low and reduced probability to flower results in prevention of vertical transmission via seed. This scenario is possible because the fungal symbiont *Epichloë* is entirely dependent on the grass and unable to switch between hosts due to predominant vertical transmission. Therefore, local adaptation of the host grass will benefit both symbiotic partners and causes natural selection acting against nonlocal host plant genotypes to also decrease performance of nonlocal *Epichloë* strains.

Microbial local adaptation to their host’s internal environment can be tested by reciprocal inoculation between host and microbe origins. Most studies on microbial local adaptation to date have been conducted on host-pathogen systems [52, 53]. In mutually beneficial symbiotic interactions, local mycorrhizal fungi have been shown to contribute to host fitness in local and nonlocal environments [8, 54]. Our present study included only plants naturally occurring with or without *Epichloë*, as this allowed determining how selection acts on natural genotype combinations in the wild. However, in this system it is also possible to grow the same host plant genotypes with and without *Epichloë* where the symbiont has been experimentally removed but requires careful control of how the removal treatment (heating seeds or fungicide application) could affect host plant fitness. Studies involving experimental inoculation of selected *Epichloë* strain in seedlings without *Epichloë*, would enable testing for different grass-*Epichloë* genotype combinations, and even three-way interactions (host genotype x *Epichloë* genotype x environment) in the wild. Also, in order to better estimate the role of production of anti-herbivore compounds in natural grass-*Epichloë* populations, studies are currently on the way to characterize alkaloid production profiles of *Epichloë* originating from different regions.

## Conclusions

Our study shows that adaptive evolution in contrasting climatic environments has resulted in local adaptation across the European range in the perennial host grass *F*. *rubra*. We found that large-scale abiotic environments did not result in strong differences in fitness between genotypes naturally occurring with or without *Epichloë* in the absence of high herbivory pressure. In the case of tight fitness linkage, however, it should be noted that selection against nonlocal host genotypes indirectly also decreases fitness of nonlocal symbiont genotypes and thus possibly contributing to the evolution of the symbiont. Future studies should strive for combining reciprocal transplantation experiments with reciprocal inoculations to unravel more complex interactions between host and symbiont genotypes and natural environments.

## Supporting information

S1 Table Number of genotypes per population. Number of genotypes per population included in the reciprocal transplant experiment of *Festuca rubra* with and without *Epichloë* symbiont.
(DOCX)

S2 Table Descriptive statistics by region. Descriptive statistics (proportion/mean ± SD) and sample size (n; number of plants and number of genotypes in brackets) by region of origin for fitness estimates at each reciprocal transplant site.
(DOCX)

S3 Table Descriptive statistics by *Epichloë* status. Descriptive statistics (proportion/mean ± SD) and sample size (n; number of plants and number of genotypes) by *Epichloë* status, region of origin for fitness estimates at each reciprocal transplant site.
(DOCX)

S4 Table Significance of fitness differences between plants with and without *Epichloë*. Results of likelihood ratio tests between generalized linear models in R testing for effect of presence of *Epichloë* in flowering propensity and biomass in second year at each reciprocal transplant site of *Festuca rubra* in northern and southern Finland, Faroe Islands and Spain.
(DOCX)

S1 Fig Phenotypic plasticity in biomass. Reaction norms for mean biomass for *Festuca rubra* genotypes across sites.
(TIF)

S2 Fig Flowering propensity in the field. Mean flowering propensity of survivors in a four-way reciprocal transplant experiment of the host plant *Festuca rubra* from northern Finland, Faroe Islands, southern Finland and Spain in their local environments in three study years.
(TIF)

S3 Fig Number of flowering culms in the field. Distribution of genotypic means for number of flowering culms for the host plant *Festuca rubra* from northern Finland, Faroe Islands, southern Finland and Spain in the first year in northern Finland, Faroe Islands and southern Finland and Spain in a four-way reciprocal transplant experiment in local environments of geographic origins.
(TIF)

S1 Data Fitness data for the reciprocal transplant experiment. Data for *Festuca rubra* genotypes at the four transplantation sites used in the analysis including explanations for the data columns
(XLSX)
