# Supplementary material for: Local adaptation in natural European host grass populations with asymmetric symbiosis
Source: PLoS One. 2019 Apr 17;14(4):e0215510. doi: 10.1371/journal.pone.0215510 (PMC6469795; doi:10.1371/journal.pone.0215510)
Supplement: S1 Table — Number of genotypes per population included in the reciprocal transplant experiment of Festuca rubra with and without Epichloë symbiont. (DOCX) [file pone.0215510.s001.docx]

**S1 Table. Number of genotypes per population.** Number of genotypes per population included in the reciprocal transplant experiment of *Festuca rubra* with and without *Epichloë* symbiont.

| Region | Population | Number of host plant genotypes | | |
| --- | --- | --- | --- | --- |
|  |  | with *Epichloë* | without *Epichloë* | Total |
| N Finland | MS1K | 12 | 11 | 23 |
|  | MS2K | 9 | 8 | 17 |
|  | Total per region | 21 | 19 | 40 |
| Faroe | FAS1 | 11 | 7 | 18 |
|  | FAS2 | 10 | 7 | 17 |
|  | Total per region | 21 | 14 | 35 |
| S Finland | HA1 | 0 | 6 | 6 |
|  | HA2 | 0 | 16 | 16 |
|  | HA3 | 0 | 10 | 10 |
|  | Total per region | 0 | 32 | 32 |
| Spain | SPGD | 5 | 3 | 8 |
|  | SPLV | 5 | 1 | 6 |
|  | SPPOR | 12 | 13 | 25 |
|  | Total per region | 22 | 17 | 39 |
