# Supplementary material for: Local adaptation in natural European host grass populations with asymmetric symbiosis
Source: PLoS One. 2019 Apr 17;14(4):e0215510. doi: 10.1371/journal.pone.0215510 (PMC6469795; doi:10.1371/journal.pone.0215510)
Supplement: S2 Table — Descriptive statistics (proportion/mean ± SD) and sample size (n; number of plants and number of genotypes in brackets) by region of origin for fitness estimates at each reciprocal transplant site. (DOCX) [file pone.0215510.s002.docx]

**S2 Table. Descriptive statistics by region.** Descriptive statistics (proportion/mean ± SD) and sample size (n; number of plants and number of genotypes in brackets) by region of origin for fitness estimates in at each reciprocal transplant site of *Festuca rubra* in northern and southern Finland, Faroe Islands and Spain.

|  |  | Transplantation site | | | | | | | |
| --- | --- | --- | --- | --- | --- | --- | --- | --- | --- |
| Survival |  | N Finland | | Faroe Islands | | S Finland | | Spain | |
| cumulative | Origin | n | Proportion of plants that survived | n | Proportion of plants that survived | n | Proportion of plants that survived | n | Proportion of plants that survived |
|  | N Finland | 115 (39) | 0.77 | 118 (40) | 0.78 | 110 (40) | 0.82 | 109 (39) | 0.24 |
|  | Faroe Islands | 79 (34) | 0.63 | 66 (33) | 0.50 | 76 (33) | 0.45 | 66 (29) | 0.39 |
|  | S Finland | 91 (31) | 0.44 | 86 (30) | 0.85 | 87 (30) | 0.72 | 85 (31) | 0.20 |
|  | Spain | 108 (37) | 0.44 | 105 (37) | 0.60 | 106 (39) | 0.56 | 94 (38) | 0.78 |
| 1st year | N Finland | 115 (39) | 0.96 | 118 (40) | 0.86 | 110 (40) | 0.98 | 109 (39) | 0.99 |
|  | Faroe Islands | 79 (34) | 0.90 | 66 (33) | 0.68 | 76 (33) | 0.82 | 66 (29) | 0.71 |
|  | S Finland | 91 (31) | 0.86 | 86 (30) | 0.95 | 87 (30) | 0.92 | 85 (31) | 0.95 |
|  | Spain | 108 (37) | 0.73 | 105 (37) | 0.87 | 106 (39) | 0.90 | 94 (38) | 0.96 |
| 2nd year | N Finland | 110 (39) | 0.95 | 102 (40) | 0.92 | 108 (40) | 0.87 | 109 (39) | 0.99 |
|  | Faroe Islands | 71 (33) | 0.85 | 45 (27) | 0.78 | 62 (30) | 0.77 | 47 (26) | 0.83 |
|  | S Finland | 78 (31) | 0.88 | 82 (30) | 0.90 | 80 (30) | 0.86 | 81 (31) | 0.73 |
|  | Spain | 79 (35) | 0.86 | 91 (36) | 0.70 | 95 (38) | 0.69 | 90 (37) | 0.92 |
| 3rd year | N Finland | 105 (39) | 0.85 | 94 (40) | 0.98 | 94 (40) | 0.96 | 108 (39) | 0.99 |
|  | Faroe Islands | 60 (30) | 0.83 | 35 (23) | 0.94 | 48 (29) | 0.71 | 39 (23) | 0.95 |
|  | S Finland | 69 (30) | 0.58 | 74 (30) | 0.99 | 69 (30) | 0.91 | 59 (27) | 0.88 |
|  | Spain | 68 (34) | 0.71 | 64 (32) | 0.98 | 66 (33) | 0.89 | 83 (37) | 0.99 |
| 4th year | N Finland | . | . | . | . | . | . | 106 (38) | 0.25 |
|  | Faroe Islands | . | . | . | . | . | . | 37 (22) | 0.70 |
|  | S Finland | . | . | . | . | . | . | 52 (24) | 0.33 |
|  | Spain | . | . | . | . | . | . | 82 (37) | 0.89 |
| Biomass |  | N Finland | | Faroe Islands | | S Finland | | Spain | |
| 1st year | Origin | n | mean ± SD | n | mean ± SD | n | mean ± SD | n | mean ± SD |
|  | N Finland | 110 (39) | 2.99±3.05 | 102 (40) | 1.53±1.5 | 107 (40) | 11.59±9.4 | 109 (39) | 21.53±14.26 |
|  | Faroe Islands | 71 (33) | 3.26±3.95 | 45 (27) | 3.24±4.95 | 62 (30) | 8.69±12.14 | 47 (26) | 11.43±16.06 |
|  | S Finland | 78 (31) | 1.49±2.34 | 82 (30) | 1.38±1.68 | 80 (30) | 7.73±7.65 | 81 (31) | 11.12±9.59 |
|  | Spain | 79 (35) | 1.15±2.91 | 91 (36) | 2.18±2.45 | 95 (38) | 4.61±6.58 | 89 (37) | 27.3±26.87 |
| 2nd year | N Finland | 103 (39) | 1.5±2.32 | 94 (40) | 5.66±8.4 | 94 (40) | 15.98±25.96 | 107 (39) | 39.42±23.31 |
|  | Faroe Islands | 60 (30) | 0.8±1.21 | 36 (23) | 12.47±22.22 | 48 (29) | 43.96±73.18 | 37 (22) | 36.06±32.97 |
|  | S Finland | 69 (30) | 0.61±1.65 | 74 (30) | 3.91±5.06 | 69 (30) | 14.36±20.92 | 58 (27) | 29.68±22.58 |
|  | Spain | 68 (34) | 0.26±0.6 | 64 (32) | 2.59±4.03 | 66 (33) | 2.25±3.47 | 83 (37) | 55.41±41.21 |
| Cumulative reproductive success |  | N Finland | | Faroe Islands | | S Finland | | Spain | |
|  | Origin | n | mean ± SD | n | mean ± SD | n | mean ± SD | n | mean ± SD |
|  | N Finland | 115 (39) | 11.06±11.99 | 118 (40) | 10.64±11.87 | 110 (40) | 38.31±67.80 | 110 (39) | 155.8±118.6 |
|  | Faroe Islands | 79 (34) | 2.66±4.78 | 66 (33) | 6.35±10.88 | 76 (33) | 15.97±42.39 | 66 (29) | 35.5±85.02 |
|  | S Finland | 91 (31) | 4.11±7.03 | 86 (30) | 16.95±22.10 | 87 (30) | 47.37±64.36 | 85 (31) | 79.76±99.27 |
|  | Spain | 108 (37) | 1.77±4.02 | 105 (37) | 11.65±15.04 | 106 (39) | 20.3±34.62 | 94 (38) | 163.1±122.7 |
| Flowering propensity | | N Finland | | Faroe Islands | | S Finland | | Spain | |
| 1st year | Origin | n | Proportion of live plants that flowered | n | Proportion of live plants that flowered | n | Proportion of live plants that flowered | n | Proportion of live plants that flowered |
|  | N Finland | 110 (39) | 0.95 | 102 (40) | 0.78 | 107 (40) | 0.87 | 109 (39) | 0.89 |
|  | Faroe Islands | 71 (33) | 0.55 | 45 (27) | 0.64 | 62 (30) | 0.50 | 47 (26) | 0.32 |
|  | S Finland | 78 (31) | 0.79 | 82 (30) | 0.72 | 80 (30) | 0.89 | 81 (31) | 0.80 |
|  | Spain | 79 (35) | 0.48 | 91 (36) | 0.91 | 95 (38) | 0.91 | 90 (37) | 0.94 |
| 2nd year | N Finland | 105 (39) | 0.61 | 94 (40) | 0.72 | 94 (40) | 0.24 | 108 (39) | 0.94 |
|  | Faroe Islands | 60 (30) | 0.08 | 36 (23) | 0.64 | 48 (29) | 0.06 | 39 (23) | 0.77 |
|  | S Finland | 69 (30) | 0.38 | 74 (30) | 0.76 | 69 (30) | 0.62 | 59 (27) | 0.98 |
|  | Spain | 68 (34) | 0.09 | 64 (32) | 0.78 | 66 (33) | 0.44 | 83 (37) | 1.00 |
| 3rd year | N Finland | 85 (38) | 0.41 | 92 (40) | 0.77 | 90 (40) | 0.72 | 107 (39) | 0.79 |
|  | Faroe Islands | 48 (27) | 0.04 | 33 (23) | 0.52 | 34 (22) | 0.71 | 37 (22) | 0.46 |
|  | S Finland | 37 (21) | 0.11 | 73 (29) | 0.86 | 63 (30) | 0.92 | 51 (24) | 0.94 |
|  | Spain | 48 (32) | 0.04 | 63 (32) | 0.22 | 59 (32) | 0.78 | 82 (37) | 1.00 |
| N flowering culms | | N Finland | | Faroe Islands | | S Finland | | Spain | |
|  |  | n | mean ± SD | n | mean ± SD | n | mean ± SD | n | mean ± SD |
| 1st year | N Finland | 105 (39) | 6.72±5.81 | 80 (38) | 4.16±3.88 | 93 (40) | 8.62±7.77 | 97 (37) | 14.48±12.01 |
|  | Faroe Islands | 39 (26) | 4.49±4.47 | 29 (20) | 4.69±3.67 | 31 (22) | 4.52±5.69 | 15 (12) | 8.73±8.54 |
|  | S Finland | 62 (29) | 4.24±3.89 | 59 (29) | 3.69±2.93 | 71 (29) | 11.83±7.6 | 65 (29) | 7.06±6.54 |
|  | Spain | 38 (27) | 4.68±5.14 | 83 (35) | 8.78±6.39 | 86 (38) | 10.74±10.04 | 85 (37) | 27.09±23.19 |
| 2nd year | N Finland | 64 (35) | 5.84±4.95 | 68 (34) | 7.43±6.71 | 23 (14) | 19.87±46.25 | 102 (38) | 154.25±106.76 |
|  | Faroe Islands | 5 (5) | 4.20±5.63 | 23 (16) | 8.30±9.12 | 3 (3) | 10.00±14.73 | 29 (20) | 76.28±110.56 |
|  | S Finland | 26 (19) | 4.08±7.41 | 56 (26) | 7.82±9.63 | 43 (25) | 13.79±21.42 | 58 (27) | 108.98±99.02 |
|  | Spain | 6 (6) | 1.83±0.75 | 50 (28) | 9.16±12.75 | 29 (17) | 6.38±10.4 | 83 (37) | 156.93±100.97 |
| 3rd year | N Finland | 35 (27) | 5.49±5.23 | 71 (35) | 5.89±5.43 | 65 (33) | 45.46±62.35 | . | . |
|  | Faroe Islands | 2 (2) | 7.00±8.49 | 17 (12) | 5.41±3.37 | 24 (16) | 43.50±61.09 | . | . |
|  | S Finland | 4 (4) | 1.25±0.5 | 63 (27) | 12.73±14.85 | 58 (30) | 46.34±56.22 | . | . |
|  | Spain | 2 (2) | 1.00±0 | 14 (12) | 2.57±2.71 | 46 (28) | 22.67±34.91 | . | . |
