# Supplementary material for: Local adaptation in natural European host grass populations with asymmetric symbiosis
Source: PLoS One. 2019 Apr 17;14(4):e0215510. doi: 10.1371/journal.pone.0215510 (PMC6469795; doi:10.1371/journal.pone.0215510)
Supplement: S3 Table — Descriptive statistics (proportion/mean ± SD) and sample size (n; number of plants and number of genotypes) by Epichloë status, region of origin for fitness estimates at each reciprocal transplant site. (DOCX) [file pone.0215510.s003.docx]

**S3 Table.** **Descriptive statistics by *Epichloë*** **status.** Descriptive statistics (proportion/mean ± SD) and sample size (n; number of plants and number of genotypes in brackets) by *Epichloë* status, region of origin for fitness estimates in at each reciprocal transplant site of *Festuca rubra* in northern and southern Finland, Faroe Islands and Spain.

| Cumulative survival | Site: N Finland | | | |
| --- | --- | --- | --- | --- |
|  | Without *Epichloë* | | With *Epichloë* | |
| Origin | n | proportion of plants that survived | n | proportion of plants that survived |
| N Finland | 53 (18) | 0.77 | 62 (21) | 0.77 |
| Faroe Islands | 30 (13) | 0.63 | 49 (21) | 0.63 |
| Spain | 48 (17) | 0.50 | 60 (20) | 0.40 |
| Cumulative reproductive success | Site: N Finland | | | |
|  | Without *Epichloë* | | With *Epichloë* | |
| Origin | n | mean ± SD | n | mean ± SD |
| N Finland | 53 (18) | 8.51 ± 7.72 | 62 (21) | 13.24 ± 14.40 |
| Faroe Islands | 30 (13) | 3.97 ± 6.59 | 49 (21) | 1.86 ± 3.01 |
| Spain | 48 (17) | 1.40 ± 3.54 | 60 (20) | 2.07 ± 4.38 |
| Biomass g/plant (2^nd^ year) | Site: N Finland | | | |
|  | Without *Epichloë* | | With *Epichloë* | |
| Origin | n | mean ± SD | n | mean ± SD |
| N Finland | 47 (18) | 1.06 ± 1.27 | 56 (21) | 1.87 ± 2.88 |
| Faroe Islands | 22 (11) | 1.31 ± 1.64 | 38 (19) | 0.51 ± 0.75 |
| Spain | 30 (15) | 0.22 ± 0.55 | 38 (19) | 0.29 ± 0.64 |
| Flowering propensity (2^nd^ year) | Site: N Finland | | | |
|  | Without *Epichloë* | | With *Epichloë* | |
| Origin | n | proportion of live plants that flowered | n | proportion of live plants that flowered |
| N Finland | 48 (18) | 0.63 | 57 (21) | 0.60 |
| Faroe Islands | 22 (11) | 0.18 | 38 (19) | 0.03 |
| Spain | 30 (15) | 0.03 | 38 (19) | 0.13 |

| Cumulative survival | Site: Faroe Islands | | | |
| --- | --- | --- | --- | --- |
|  | Without *Epichloë* | | With *Epichloë* | |
| Origin | n | proportion of plants that survived | n | proportion of plants that survived |
| N Finland | 56 (19) | 0.75 | 62 (21) | 0.81 |
| Faroe Islands | 28 (14) | 0.39 | 38 (19) | 0.58 |
| Spain | 47 (17) | 0.62 | 58 (20) | 0.59 |
| Cumulative reproductive success | Site: Faroe Islands | | | |
|  | Without *Epichloë* | | With *Epichloë* | |
| Origin | n | mean ± SD | n | mean ± SD |
| N Finland | 56 (19) | 11.07 ± 12.21 | 62 (21) | 10.26 ± 11.65 |
| Faroe Islands | 28 (14) | 4.89 ± 8.21 | 38 (19) | 7.42 ± 12.49 |
| Spain | 47 (17) | 11.91 ± 19.08 | 58 (20) | 11.43 ± 10.91 |
| Biomass g/plant (2^nd^ year) | Site: Faroe Islands | | | |
| Origin | Without *Epichloë* | | With *Epichloë* | |
|  | n | mean ± SD | n | mean ± SD |
| N Finland | 43 (19) | 4.89 ± 4.90 | 51 (21) | 6.31 ± 10.49 |
| Faroe Islands | 12 (8) | 12.04 ± 25.31 | 24 (15) | 12.68 ± 21.10 |
| Spain | 29 (16) | 3.69 ± 5.49 | 35 (16) | 1.68 ± 1.83 |
| Flowering propensity (2^nd^ year) | Site: Faroe Islands | | | |
| Origin | Without *Epichloë* | | With *Epichloë* | |
|  | n | proportion of live plants that flowered | n | proportion of live plants that flowered |
| N Finland | 43 (19) | 0.70 | 51 (21) | 0.75 |
| Faroe Islands | 12 (8) | 0.58 | 24 (15) | 0.67 |
| Spain | 29 (16) | 0.79 | 35 (16) | 0.77 |

| Cumulative survival | Site: S Finland | | | |
| --- | --- | --- | --- | --- |
|  | Without *Epichloë* | | With *Epichloë* | |
| Origin | n | proportion of plants that survived | n | proportion of plants that survived |
| N Finland | 52 (19) | 0.79 | 58 (21) | 0.84 |
| Faroe Islands | 31 (13) | 0.45 | 45 (20) | 0.44 |
| Spain | 45 (17) | 0.51 | 61 (22) | 0.59 |
| Cumulative reproductive success | Site: S Finland | | | |
|  | Without *Epichloë* | | With *Epichloë* | |
| Origin | n | mean ± SD | n | mean ± SD |
| N Finland | 52 (19) | 17.04 ± 28.17 | 58 (21) | 57.38 ± 85.43 |
| Faroe Islands | 31 (13) | 19.74 ± 59.61 | 45 (20) | 13.38 ± 24.97 |
| Spain | 45 (17) | 16.18 ± 26.92 | 61 (22) | 23.34 ± 39.29 |
| Biomass g/plant (2^nd^ year) | Site: S Finland | | | |
| Origin | Without *Epichloë* | | With *Epichloë* | |
|  | n | mean ± SD | n | mean ± SD |
| N Finland | 42 (19) | 8.75 ± 12.44 | 52 (21) | 21.82 ± 32.04 |
| Faroe Islands | 18 (11) | 46.85 ± 75.61 | 30 (18) | 42.22 ± 72.93 |
| Spain | 24 (14) | 2.02 ± 2.93 | 42 (19) | 2.38 ± 3.78 |
| Flowering propensity (2^nd^ year) | Site: S Finland | | | |
| Origin | Without *Epichloë* | | With *Epichloë* | |
|  | n | proportion of live plants that flowered | n | proportion of live plants that flowered |
| N Finland | 42 (19) | 0.05 | 52 (21) | 0.40 |
| Faroe Islands | 18 (11) | 0.06 | 30 (18) | 0.07 |
| Spain | 24 (14) | 0.29 | 42 (19) | 0.52 |

| Cumulative survival | Site: Spain | | | |
| --- | --- | --- | --- | --- |
|  | Without *Epichloë* | | With *Epichloë* | |
| Origin | n | proportion of plants that survived | n | proportion of plants that survived |
| N Finland | 52 (18) | 0.25 | 57 (21) | 0.23 |
| Faroe Islands | 22 (10) | 0.23 | 44 (19) | 0.48 |
| Spain | 43 (17) | 0.81 | 51 (21) | 0.75 |
| Cumulative reproductive success | Site: Spain | | | |
|  | Without *Epichloë* | | With *Epichloë* | |
| Origin | n | mean ± SD | n | mean± SD |
| N Finland | 52 (18) | 153.77 ± 123.23 | 58 (21) | 157.64 ± 115.24 |
| Faroe Islands | 22 (10) | 21.09 ± 45.80 | 44 (19) | 42.70 ± 98.71 |
| Spain | 43 (17) | 166.44 ± 128.29 | 51 (21) | 160.22 ± 118.99 |
| Biomass g/plant (2^nd^ year) | Site: Spain | | | |
| Origin | Without *Epichloë* | | With *Epichloë* | |
|  | n | mean ± SD | n | mean ± SD |
| N Finland | 51 (18) | 35.22 ± 24.27 | 56 (21) | 43.25 ± 21.91 |
| Faroe Islands | 9 (5) | 25.74 ± 26.34 | 28 (17) | 39.38 ± 34.59 |
| Spain | 40 (17) | 58.60 ± 52.23 | 43 (20) | 52.45 ± 27.63 |
| Flowering propensity (2^nd^ year) | Site: Spain | | | |
| Origin | Without *Epichloë* | | With *Epichloë* | |
|  | n | proportion of live plants that flowered | n | proportion of live plants that flowered |
| N Finland | 51 (18) | 0.90 | 57 (21) | 0.98 |
| Faroe Islands | 11 (6) | 0.82 | 28 (17) | 0.75 |
| Spain | 40 (17) | 1.00 | 43 (20) | 1.00 |
