# Supplementary material for: Local adaptation in natural European host grass populations with asymmetric symbiosis
Source: PLoS One. 2019 Apr 17;14(4):e0215510. doi: 10.1371/journal.pone.0215510 (PMC6469795; doi:10.1371/journal.pone.0215510)
Supplement: S4 Table — Results of likelihood ratio tests between generalized linear models in R testing for effect of presence of Epichloë in flowering propensity and biomass in second year at each reciprocal transplant site of Festuca rubra in northern and southern Finland, Faroe Islands and Spain. (DOCX) [file pone.0215510.s004.docx]

**S4 Table. Significance of fitness differences between plants with and without *Epichloë*.** Results of likelihood ratio tests (deviance and P-value; d.f. = 1) between generalized linear models in R testing for effect of presence of *Epichloë* in flowering propensity and biomass in second year at each reciprocal transplant site of *Festuca rubra* in northern and southern Finland, Faroe Islands and Spain.

|  |  | Transplantation site | | | | | | | | | | |
| --- | --- | --- | --- | --- | --- | --- | --- | --- | --- | --- | --- | --- |
|  |  | N Finland | |  | Faroe Islands | |  | S Finland | |  | Spain | |
| Fitness estimate and region of origin |  | Deviance | P |  | Deviance | P |  | Deviance | P |  | Deviance | P |
| Cumulative survival |  |  |  |  |  |  |  |  |  |  |  |  |
| N Finland |  | 0.00 | 0.9938 |  | 0.55 | 0.4602 |  | 0.58 | 0.4444 |  | 0.07 | 0.7885 |
| Faroe Islands |  | 0.00 | 0.9951 |  | 2.25 | 0.1339 |  | 0.00 | 0.9508 |  | **4.01** | **0.0451** |
| Spain |  | 1.08 | 0.2987 |  | 0.10 | 0.7485 |  | 0.66 | 0.4182 |  | 0.64 | 0.4224 |
| Cumulative reproductive success |  |  |  |  |  |  |  |  |  |  |  |  |
| N Finland |  | 0.57 | 0.2600 |  | 0.04 | 0.8267 |  | **6.32** | **0.0445** |  | 0.67 | 0.4860 |
| Faroe Islands |  | 1.04 | 0.1887 |  | 0.26 | 0.6752 |  | 0.46 | 0.6498 |  | 3.55 | 0.3040 |
| Spain |  | 0.65 | 0.2715 |  | 0.00 | 0.9611 |  | 1.89 | 0.2005 |  | 0.34 | 0.5616 |
| Biomass (2^nd^ year) |  |  |  |  |  |  |  |  |  |  |  |  |
| N Finland |  | 2.65 | 0.0607 |  | 0.76 | 0.3046 |  | **5.62** | **0.0465** |  | 0.49 | 0.1596 |
| Faroe Islands |  | **8.05** | **0.0051** |  | 0.32 | 0.7317 |  | 0.00 | 0.9990 |  | 0.53 | 0.3757 |
| Spain |  | 0.23 | 0.7887 |  | 3.53 | 0.0692 |  | 0.36 | 0.6052 |  | 0.07 | 0.6724 |
| Flowering propensity (2^nd^ year) |  |  |  |  |  |  |  |  |  |  |  |  |
| N Finland |  | 0.09 | 0.7654 |  | 0.26 | 0.6090 |  | **18.37** | **< 0.0001** |  | 3.56 | 0.0592 |
| Faroe Islands |  | **4.31** | **0.0379** |  | 0.24 | 0.6252 |  | 0.02 | 0.8767 |  | 0.21 | 0.6436 |
| Spain |  | 2.22 | 0.1357 |  | 0.04 | 0.8344 |  | 3.42 | 0.0644 |  | 0.00 | 1.0000 |
