# Supplementary figures and images for: Local adaptation in natural European host grass populations with asymmetric symbiosis
Source: PLoS One. 2019 Apr 17;14(4):e0215510. doi: 10.1371/journal.pone.0215510 (PMC6469795; doi:10.1371/journal.pone.0215510)

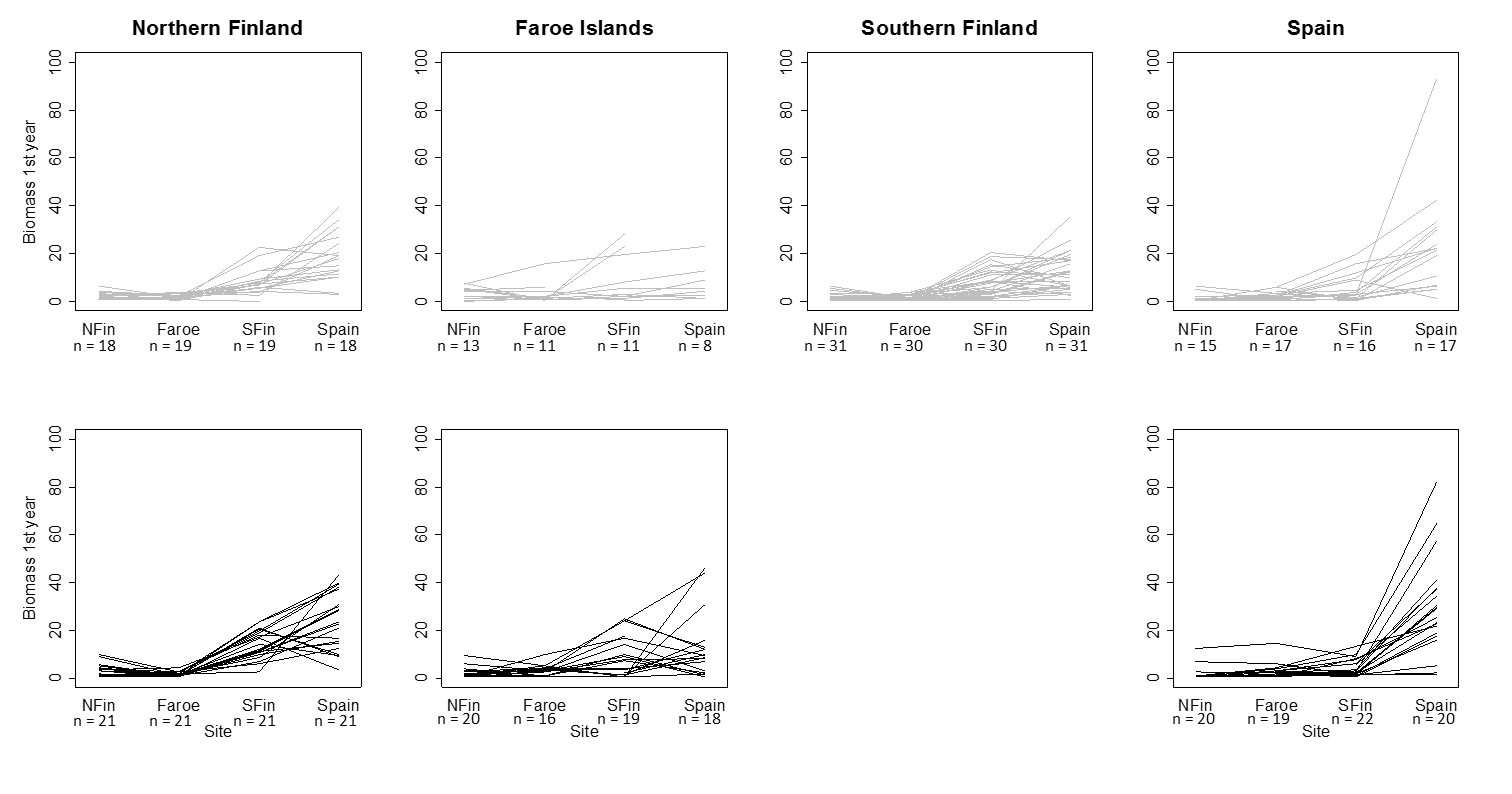

Supplement: S1 Fig — Reaction norms for mean biomass for Festuca rubra genotypes across sites. (TIF) [file pone.0215510.s005.tif]

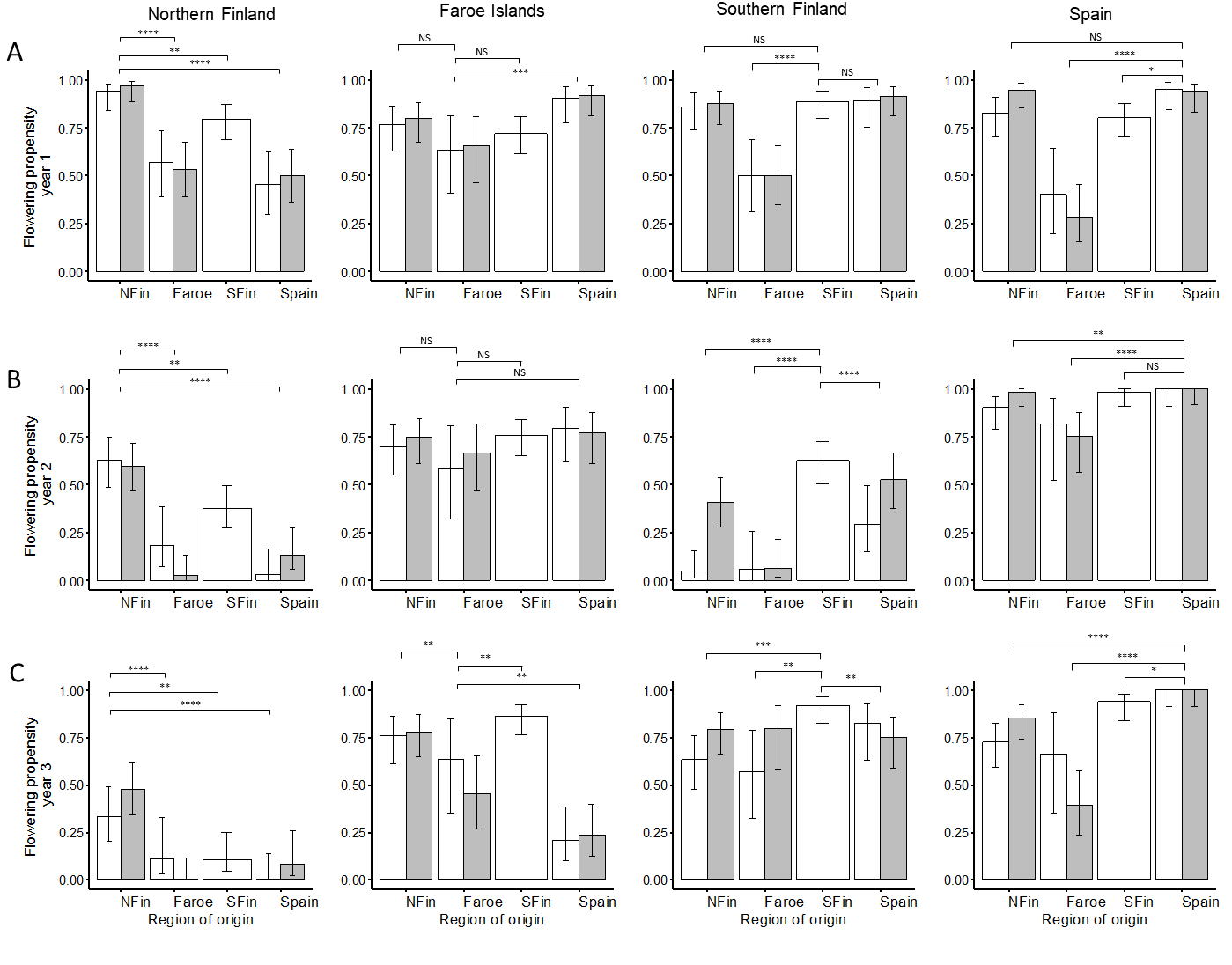

Supplement: S2 Fig — Mean flowering propensity of survivors in a four-way reciprocal transplant experiment of the host plant Festuca rubra from northern Finland, Faroe Islands, southern Finland and Spain in their local environments in three study years. (TIF) [file pone.0215510.s006.tif]

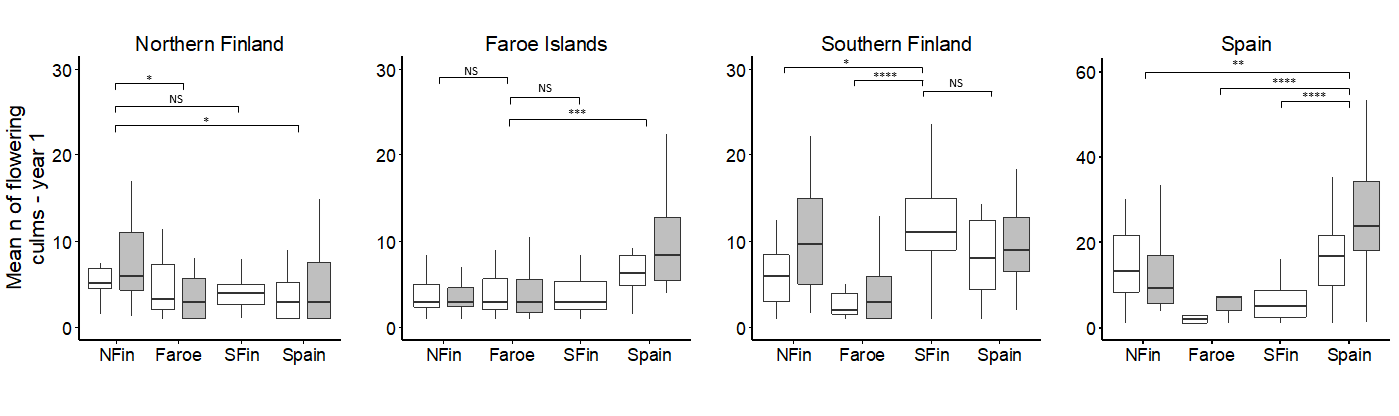

Supplement: S3 Fig — Distribution of genotypic means for number of flowering culms for the host plant Festuca rubra from northern Finland, Faroe Islands, southern Finland and Spain in the first year in northern Finland, Faroe Islands and southern Finland and Spain in a four-way reciprocal transplant experiment in local environments of geographic origins. (TIF) [file pone.0215510.s007.tif]
